# Retention of uninfected red blood cells causing congestive splenomegaly is the major mechanism of anemia in malaria

**DOI:** 10.1002/ajh.27152

**Published:** 2023-11-27

**Authors:** Steven Kho, Nurjati C. Siregar, Labibah Qotrunnada, Aurélie Fricot, Abdoulaye Sissoko, Putu A. I. Shanti, Freis Candrawati, Noy N. Kambuaya, Hasrini Rini, Benediktus Andries, David Hardy, Nur I. Margyaningsih, Fauziyah Fadllan, Desandra A. Rahmayenti, Agatha M. Puspitasari, Amelia R. Aisah, Leo Leonardo, Bagus T. G. Yayang, Dewi S. Margayani, Pak Prayoga, Leily Trianty, Enny Kenangalem, Ric N. Price, Tsin W. Yeo, Gabriela Minigo, Rintis Noviyanti, Jeanne R. Poespoprodjo, Nicholas M. Anstey, Pierre A. Buffet

**Affiliations:** ^1^ Global and Tropical Health Division Menzies School of Health Research and Charles Darwin University Darwin Northern Territory Australia; ^2^ Timika Malaria Research Program Papuan Health and Community Development Foundation Timika Indonesia; ^3^ Eijkman Institute for Molecular Biology Jakarta Indonesia; ^4^ Department of Anatomical Pathology Rumah Sakit Cipto Mangunkusumo and Universitas Indonesia Jakarta Indonesia; ^5^ UMR_S1134, BIGR, Inserm, Université de Paris Paris France; ^6^ Rumah Sakit Umum Daerah Kabupaten Mimika Timika Indonesia; ^7^ Institut Pasteur Experimental Neuropathology Unit Paris France; ^8^ Lee Kong Chian School of Medicine Nanyang Technology University Singapore Singapore; ^9^ Department of Pediatrics University of Gadjah Mada Yogyakarta Indonesia

## Abstract

Splenomegaly frequently occurs in patients with *Plasmodium falciparum* (Pf) or *P. vivax* (Pv) malarial anemia, but mechanisms underlying this co‐occurrence are unclear. In malaria‐endemic Papua, Indonesia, we prospectively analyzed red blood cell (RBC) concentrations in the spleen and spleen‐mimetic retention in 37 subjects splenectomized for trauma or hyperreactive splenomegaly, most of whom were infected with *Plasmodium*. Splenomegaly (median 357 g [range: 80–1918 g]) was correlated positively with the proportion of red‐pulp on histological sections (median 88.1% [range: 74%–99.4%]; *r* = .59, *p* = .0003) and correlated negatively with the proportion of white‐pulp (median 8.3% [range: 0.4%–22.9%]; *r* = −.50, *p* = .002). The number of RBC per microscopic field (>95% uninfected) was correlated positively with spleen weight in both Pf‐infected (*r* = .73; *p* = .017) and Pv‐infected spleens (*r* = .94; *p* = .006). The median estimated proportion of total‐body RBCs retained in Pf‐infected spleens was 8.2% (range: 1.0%–33.6%), significantly higher than in Pv‐infected (2.6% [range: 0.6%–23.8%]; *p* = .015) and PCR‐negative subjects (2.5% [range: 1.0%–3.3%]; *p* = .006). Retained RBCs accounted for over half of circulating RBC loss seen in Pf infections. The proportion of total‐body RBC retained in Pf‐ and Pv‐infected spleens correlated negatively with hemoglobin concentrations (*r* = −.56, *p* = .0003), hematocrit (*r* = −.58, *p* = .0002), and circulating RBC counts (*r* = −.56, *p* = .0003). Splenic CD71‐positive reticulocyte concentrations correlated with spleen weight in Pf (r = 1.0; *p* = .003). Retention rates of peripheral and splenic RBCs were correlated negatively with circulating RBC counts (*r* = −.69, *p* = .07 and *r* = −.83, *p* = .008, respectively). In conclusion, retention of mostly uninfected RBC in the spleen, leading to marked congestion of the red‐pulp, was associated with splenomegaly and is the major mechanism of anemia in subjects infected with *Plasmodium*, particularly Pf.

## 1 INTRODUCTION

Malaria remains a major global health problem with an estimated 247 million cases worldwide in 2021 and many more residents of endemic areas having asymptomatic parasitemia (chronic malaria).^1^ Severe anemia is the most frequent severe form of malaria with mild–moderate anemia common in chronic malaria.^1^, ^2^ Markers of impaired red blood cell (RBC) production or excessive RBC loss are observed in malaria,^3^, ^4^ but the respective contributions of these mechanisms to anemia have never been quantitatively determined. It has been hypothesized that impaired production may predominate in chronic infections, while RBC loss, in part due to splenic RBC clearance, may play a greater role in acute episodes.^5^ Splenomegaly is a common manifestation in malaria‐endemic areas and occurs in patients infected with either *P. falciparum* (Pf) or *P. vivax* (Pv).^6^ In patients with malaria, the presence of splenomegaly is correlated with anemia,^7^, ^8^, ^9^, ^10^, ^11^, ^12^ but whether the enlarged spleen is a marker of more severe or more prolonged infection (both potentially associated with more profound anemia) or a direct mediator of anemia is unknown. Because human spleen studies are ethically and logistically demanding, quantifying the relationship between spleen size, splenic architecture, RBC composition, and malarial anemia has not been previously performed.

In 37 patients undergoing splenectomy (mostly for trauma) in malaria‐endemic Papua, 32 were infected with malaria parasites and 26 were anemic. We examined their splenic tissue architecture and accumulation and subpopulation composition of RBC to determine the predominant mechanisms underlying malarial splenomegaly. We also compared retention characteristics of RBC from splenic and circulating blood to determine the direct contribution of the spleen to anemia in malaria.

## 2 METHODS

### 2.1 Study participants and samples

Timika, Papua, Indonesia, is a lowland forest region with high and unstable transmission of Pf and Pv.^2^ We prospectively enrolled consecutive patients undergoing splenectomy at the Timika regional hospital for any clinical indication between 2015–17 (Cohort 1) and 2019–21 (Cohort 2). Previously published protocols for enrolment and clinical management in Cohort 1^13^, ^14^ were adopted for patients enrolled in Cohort 2.

Spleen tissue was collected from the operating theater during surgery, then rapidly processed for sliced spleen blood, and stored as snap‐frozen biopsies and formalin‐fixed paraffin‐embedded (FFPE) blocks, as described previously.^13^, ^14^ Spleen tissue sections of 5 μm thickness were prepared from FFPE‐blocks for histological and immunohistochemical staining. Peripheral venous blood samples were collected intraoperatively for automated blood counts, peripheral blood smears, and fresh assays and stored as frozen‐packed RBCs and heparinized plasma. Clinical and demographic data were recorded.

### 2.2 *Plasmodium* detection

Detailed methods for the detection of *Plasmodium* infection are described.^13^, ^14^ Briefly, spleen tissue sections and peripheral blood smears were Giemsa‐stained and examined for malaria parasites by optical microscopy. QIAamp DNA mini‐kits (Qiagen, Germany) were used to extract DNA from frozen spleen biopsies and peripheral RBCs using a modified protocol, and then, genomic DNA was examined for *Plasmodium* by PCR.^14^

### 2.3 Splenic architectural analyses

Giemsa‐stained spleen sections were scanned at 400x magnification on an AxioScan Z1 (Carl Zeiss, Germany) or Aperio AT2 (Leica Biosystems, Wetzlar, Germany). Using software tracing tools (which provide the area in μm^2^ of customized regions), the total section area and zones of white‐pulp and non‐white‐pulp structures (capsule, large vessels, central artery, trabeculus, and hilus) were quantified by a research microscopist for each sample, blinded to patient number and *Plasmodium* infection. Circulatory spaces (perifollicular zones) around the follicles were not considered white‐pulp in this analysis. Red‐pulp area was calculated by subtracting white‐pulp and non‐white‐pulp structures from the total section area. The percentages of white‐pulp and red‐pulp were calculated for each spleen and categorized by infecting *Plasmodium* species and spleen weight. The number of RBCs/high‐powered field (HPF) was counted in 10 red‐pulp HPFs on Giemsa‐stained spleen sections.

### 2.4 Intrasplenic and circulating RBC loads

**FIGURE 1:**
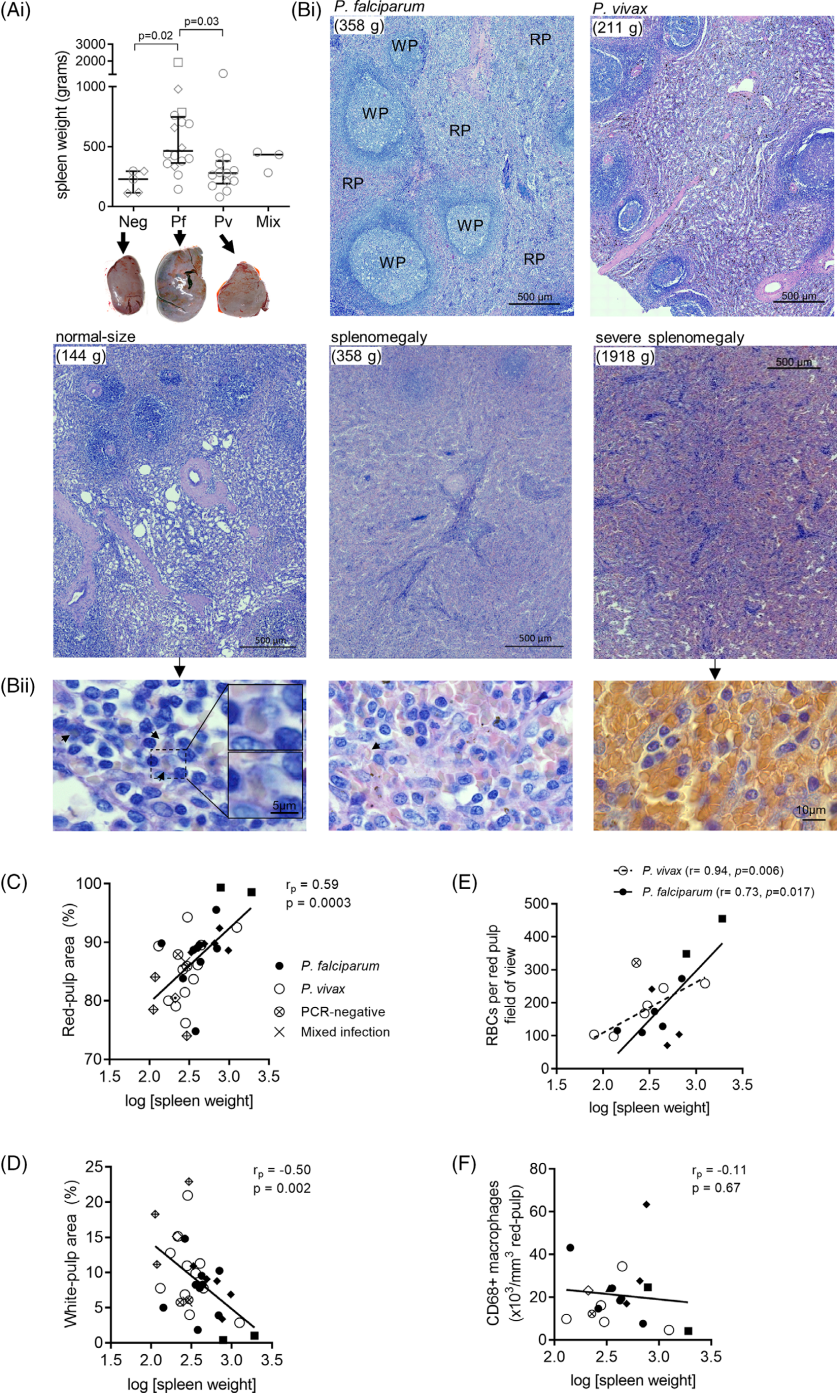
Architectural associations with spleen weight. The weight of each spleen was recorded (to the closest gram) and grouped according to infecting *Plasmodium* species comprising of 16 with Pf, 13 with Pv, three mixed Pf–Pv infection, and five PCR‐negative endemic controls (A). Representative images of spleens are shown below each group (except mixed infections). Giemsa‐stained spleen sections were scanned at 400x magnification on a Carl Zeiss AxioScan Z1 or Leica Aperio AT2 to determine the percentage of area covered by white‐pulp and red‐pulp. Representative histological images from *P. falciparum‐* and *P. vivax*‐infected spleens are presented, as well as images from cases of splenomegaly and severe splenomegaly with *P. falciparum* infection (Bi). Representative histological illustrations of the degree of RBC congestion in the red‐pulp across difference spleen sizes are also provided (Bii, arrowheads indicate erythrophagocytosis). Correlations between spleen weight and red‐pulp (C) and spleen weight and white‐pulp (D) were determined using the Pearson test. The number of RBCs per red‐pulp field of view was counted in a subset of *P. falciparum* (*n* = 10), *P. vivax* (*n* = 6), and PCR‐negative (*n* = 1) spleens and correlated with spleen weight (E). The number of macrophages (CD68+ cells) per unit volume of red‐pulp was also counted in a subset of spleens (*n* = 19) and correlated with spleen weight (F). In A, median, interquartile range, and individual datapoints are shown and compared using the Kruskal–Wallis test with Dunn's multiple comparison. In C–F, spleen weights were log‐transformed and line of best fit was determined by linear regression. A p‐value <0.05 was considered significant. Diamond datapoints represent spleens from patients recently treated with antimalarials, circular datapoints represent spleens from untreated patients, and square datapoints represent patients with suspected hyperreactive malarial splenomegaly. Pf, *Plasmodium falciparum*; Pv, *Plasmodium vivax*; RBC, red blood cell; PCR, polymerase chain reaction. [Color figure can be viewed at wileyonlinelibrary.com]

A simple categorical approach was used to determine intrasplenic RBC loads accurately based on spleen weight and experimental data. An internal control experiment was performed on four spleens from patients undergoing left spleno‐pancreatectomy in France, a non‐malaria‐endemic area, to determine the volume of packed RBC inside a normal adult human spleen. Briefly, the four spleens were weighed and then flushed with 100 to 500 mL of Krebs‐albumin solution^15^ using a 3‐mm catheter inserted into the artery, with several wash fractions collected consecutively from the vein into 5‐mL lithium‐heparin blood tubes (first venous effluent) or 50‐mL polyethylene tubes (subsequent rinsing steps). After centrifugation for 5 minutes at 1500 G, the volume of the RBC pellet was measured in each tube using P1000 to P20 pipettors. Hematocrit in the last rinsing 50‐mL tube was <0.1% (RBC pellet <50 μL) in all cases. The mean (±standard deviation) total volume of RBC pellet in a normal spleen was 10.1 ± 2.8 mL, and when normalized to spleen weight, it was 8.3 ± 3.7 mL/100 grams of tissue (Table S1). Given that splenic blood hematocrit is considered to be twice that of circulating blood,^16^ we assumed a spleen blood hematocrit of 60% in normal spleens and that splenomegaly‐related RBC congestion (Figure 1E) increases spleen blood hematocrit in larger spleens. Using these data and assumptions, we generated a simple categorical approach to estimate the volume of intrasplenic RBCs, circulating RBCs, and the proportion of total‐body (circulating plus splenic) RBCs retained in the spleen in our cohort:

**Table:** 

Spleen weight (grams)	Spleen blood volume per 100 g tissue (L)	Spleen blood hematocrit (%)	Spleen RBC volume per 100 g tissue (L)
0–200	15	60	9
201–400	20	65	13
401–800	25	70	17.5
>800	30	75	22.5

Using the above, the volume of intrasplenic RBCs (in mL) was calculated for each spleen as follows:

$$=\frac{\left[\text{spleen weight in}\;g\right]}{100}\times \left[\begin{array}{c} \text{spleen}\;\mathrm{RBC}\;\text{volume}\;\left(\text{in}\;\mathrm{mL}\right)\;\\ \mathrm{per}\;100\;g\;\text{tissue} \end{array}\right].$$

The volume of circulating RBC (in L) was calculated for each individual as follows:

$$=\left[\text{circulating blood volume in}\;L\right]\times \frac{\left[\text{circulating blood hematocrit}\%\right]}{100}.$$

For each individual, circulating blood volumes were calculated using Nadler's method^14^ and circulating blood hematocrit values were obtained from an automated analyzer.

The proportion of total‐body (circulating plus splenic) RBCs that are being retained in the spleen was calculated as follows:

$$=\frac{\left[\text{volume of intrasplenic RBCs}\right]}{\left[\text{volume of intrasplenic RBCs}\right]+\left[\text{volume of circulating RBCs}\right]}\times 100.$$

### 2.5 Macrophage immunohistochemistry

Rehydrated FFPE spleen sections from Cohort 1 were stained with anti‐human CD68 (clone KP‐1, Roche, Basel, Switzerland), followed by secondary labeling with N‐Histofine (Nichirei Biosciences, Tokyo, Japan). Immunostaining procedures were performed using an automated Leica BOND‐III platform (Leica Biosystems, Wetzlar, Germany) and then counterstained with hematoxylin prior to sealing. Slides were scanned using an AxioScan Z1 (Carl Zeiss, Germany), and CD68^+^ cells were counted by a research microscopist using ImageJ (National Institute of Health, WI, USA) in a minimum area of 1 mm^2^ of red‐pulp. To determine the number of cells per unit volume (mm^3^) of red‐pulp, the number of cells per unit area (mm^2^) was multiplied by the section thickness (5 μm = 0.005 mm).

### 2.6 Ex vivo RBC retention rate

A microsphere‐based filtration of RBC (microsphiltration) assay mimicking splenic biomechanical retention was performed on a subset of acid citrate dextrose (ACD)‐anticoagulated peripheral blood and sliced spleen blood samples from nine individuals within 24 h of surgery, and RBC retention rate was calculated as previously reported.^14^, ^17^ Briefly, fresh normal human RBCs were obtained on the day of experiments and used for preparation of heated and normal control RBCs. WBC‐depleted patient samples and normal control RBCs were labeled with PKH‐26 and PKH‐67 Fluorescent Cell Linker kits (Sigma‐Aldrich, Missouri, USA), respectively, and then mixed with unlabeled normal control RBCs resulting in a single suspension containing 5% labeled patient RBCs, 5% labeled normal control RBCs, and 90% unlabeled normal control RBCs. The resulting suspension was adjusted to 2% hematocrit using 1% Albumax‐II/PBS and introduced upstream of rehydrated tips containing a microsphere layer closely resembling splenic interendothelial spaces. A total of 6 mL of flow solution was perfused through the system to facilitate filtration, with deformable cells eluting through the tips and cells below the deformability threshold retained at the microsphere layer. Each patient sample was run in triplicate, and the downstream samples were retrieved into falcon tubes. For quality control of tips, the above was performed with heated RBCs (50°C for 20 min) in place of patient RBCs as a positive control for rigid cells.

Fifty μL of each upstream and downstream sample was diluted with 450 μL of flow solution and ran on a BD Accuri C6 flow cytometer (BD Biosciences, Australia). At least 300 000 events were collected per sample. The percentage of labeled cells was gated (Figure S1) and compared in upstream and downstream suspensions, providing a measure of the rate of RBC retention, which was calculated as follows:

$$\mathrm{RBC}\;\text{retention rate}=\frac{\left[\%\text{upstream gate}\right]-\left[\%\text{downstream gate}\right]}{\left[\%\text{upstream gate}\right]}\times 100.$$

Microsphiltration was repeated on peripheral blood and sliced spleen blood from eight non‐endemic controls in France using a published high‐throughput protocol^18^ with minor modifications to the upstream sample content consisting of 20% positive control RBCs, 20% negative control RBCs, 20% patient circulating RBCs, and 20% patient splenic RBCs, with the resulting suspension introduced into filtration plates at 2% hematocrit.

### 2.7 Reticulocytes

The concentration of total circulating reticulocytes, a proxy for bone marrow function,^19^ and splenic reticulocytes categorized into CD71^−^ (mature), CD71^low^, CD71^intermediate^, and CD71^high^ was measured by fresh flow cytometry in a subset of 11 Indonesian patients as published previously.^14^ Immature CD71^+^ reticulocyte populations, used as a marker of rigid RBC,^14^, ^20^ were correlated with spleen weight as an additional measure of relationships between splenic filtration stringency and spleen size.

### 2.8 Serology

Total immunoglobulin M (IgM) levels were measured in frozen heparinized plasma samples using a BN‐ProSpec protein analyzer (Siemens, Germany). Baseline IgM levels in Timika were determined by analysis of 54 adult plasma samples from a household survey.^2^, ^14^

### 2.9 Statistics

Data analyses were performed using GraphPad Prism 9 (GraphPad, California), Stata v13 (StataCorp, USA), FlowJo (BD, Ashland), ZEN (Carl Zeiss, Germany), and Aperio ImageScope (Leica Biosystems, Wetzlar, Germany). The entirety of some images was adjusted for brightness/contrast without altering any original features. The Mann–Whitney test was used for comparison of two groups, and the Kruskal–Wallis test with Dunn's multiple comparison was used for comparisons of multiple groups. Correlations were analyzed by the Pearson or Spearman test. Partial correlation was performed in Stata (pcorr) to control for RBC counts in the association between reticulocyte concentrations and spleen weight. The chi‐squared test was used for comparison of categorical variables and population proportions.

## 3 RESULTS

### 3.1 Study cohort

Thirty‐seven consecutive patients undergoing splenectomy were enrolled (22 from Cohort 1^13,14^ and 15 from Cohort 2); 13 were infected with Pv, 16 with Pf, three with mixed Pf–Pv, and five were PCR‐negative for *Plasmodium*. Of the 36 patients who were afebrile at the time of splenectomy, eight (one Pv, four Pf, and three PCR‐negative) received antimalarial treatment in the month prior to splenectomy. One patient was acutely febrile and considered to have acute falciparum malaria at the time of splenectomy. Demographic and laboratory data from each group are given in Table 1.

**TABLE 1:** Baseline characteristics of splenectomy patients in Timika, Indonesia.

Parameters	All splenectomy patients *n* = 37	Subgroups					* *P. vivax* versus *P. falciparum* subgroup comparison *p*‐value
		*Asymptomatic P. vivax n* = 13	*Asymptomatic P. falciparum n* = 15	Asymptomatic mixed *P. vivax‐P. falciparum n* = 3	PCR‐negative control *n* = 5	Acute *P. falciparum* malaria, *n* = 1	
Age in years (median [IQR])	22 (18.5–33.5)	25 (17.5–35.5)	20 (16–40)	26 (22–28)	19 (11.5–27)	19	.99
Gender (n/N of males, [%])	28/37 (75.7)	8/13 (61.5)	11/15 (73.3)	3/3 (100)	5/5 (100)	1	.51
Ethnicity (n/N of Papuans, [%])	21/37 (56.8)	6/13 (46.2)	11/15 (73.3)	2/3 (66.7)	1/5 (20)	1	.15
Splenectomy due to trauma (n/N, %)	35/37 (94.6)	12/13 (92.3)	14/15 (93.3)	0	0	1	.92
Splenectomy due to splenomegaly (n/N, %)	2/37 (5.4)	1/13 (7.7)	1/15 (6.7)	0	0	0	.92
Body temp. in °C (median [IQR])	36.5 (36.1–36.8)	36.6 (36.3–37)	36.4 (36–36.7)	36 (36–36.2)	36.7 (36.5–37.3)	37.6	.16
Prior transfusion, (n/N, %)	10/37 (27)	4/13 (30.8)	4/15 (26.7)	0	2/5 (40)	0	.81
Hemoglobin in g/dL (median [IQR])	10.8 (8.9–12.4)	11.3 (9.2–14.2)	10.2 (7.1–10.9)	12.4 (11.8–12.5)	11.1 (8.9–13.6)	11.2	.053
Hematocrit in % (median [IQR])	32 (26.1–35.4)	33.6 (28.1–40.4)	28.8 (22.5–32.6)	35.6 (32.6–36.1)	33.0 (27.0–38.4)	31.5	**.047**
Circulating red blood cells ×10^6^/μL (median [IQR])	3.8 (3.4–4.6)	4.2 (3.6–5.0)	3.4 (3.0–3.8)	4.3 (4.2–4.6)	3.9 (3.6–4.9)	3.7	**.042**
Red blood cell distribution width—SD (fL) (median [IQR])	38.8 (37.2–43.8)	37.9 (36.6–39.7)	41.9 (37.3–47.6)	40 (34.6–52)	38.7 (35.7–39.5)	39.8	.053
Red cell distribution width—CV (%) (median [IQR])	14.1 (13–16)	13.8 (13–14.3)	15.6 (13.5–19.1)	14.3 (12.0–17.8)	13 (12.8–15.1)	12.9	**.016**
Circulating white blood cells ×10^3^/μL (median [IQR])	13.2 (7.3–18.7)	10.5 (8.1–18.2)	8.9 (5.5–13.4)	20.1 (13.7–27.5)	23.4 (17.2–29.3)	7.4	.20
Circulating lymphocytes ×10^3^/μL (median [IQR])	2.6 (1.4–4.0)	3.1 (1.7–6.7)	1.7 (1.4–3.1)	2.9 (1.2–21.7)	4.3 (2.5–5.6)	1.1	.08
Circulating monocytes ×10^3^/μL (median [IQR])	0.7 (0.6–1.1)	0.6 (0.4–0.8)	0.6 (0.6–0.8)	1.1 (1.1–1.9)	1.4 (0.9–1.8)	1.4	.57
Circulating neutrophils ×10^3^/μL (median [IQR])	8.1 (4.3–14.2)	6.8 (4.5–10.3)	4.8 (3.2–12.6)	17.7 (8.4–21.7)	13.7 (11.7–23.6)	4.9	.41
Circulating eosinophils ×10^3^/μL (median [IQR])	0.2 (0.1–0.7)	0.5 (0.1–0.9)	0.2 (0.1–0.3)	0.1 (0.1–1.1)	0.1 (0.1–0.3)	0.1	.19
Circulating platelets ×10^3^/μL (median [IQR])	207 (130–261)	259 (185.5–279.5)	134 (96–200)	207 (180–246)	224 (181–264.5)	90	**.004**
Total plasma Immunoglobulin M mg/dL (median [IQR])	188 (86–332)	104 (69–193)	306 (174–387)	452 (244–616)	108 (91–471)	110	**.004**
^a^Peripheral *Plasmodium* positive (n/N of positive [%])	29/37 (78.4)	13/13 (100)	13/15 (86.7)	3 (100)	0	1	.09
^a^Spleen *Plasmodium* positive (n/N of positive [%])	31/37 (83.8)	13/13 (100)	15/15 (100)	3(100)	0	1	.35
^b^Treated with antimalarials in the last month (n/N, [%])	11/37 (29.7)	2/13 (15.4)	4/15 (26.7)	0	3/5 (60)	2/5 (40)	.48
Red‐pulp area % (median [IQR])	88.1 (83.1–89.8)	84.5 (80.2–89.5)	89.5 (88.3–92.4)	86.7	84.1 (76.3–86.9)	89.1	**.047**
White‐pulp area % (median [IQR])	8.3 (5.6–11.2)	10.4 (7.1–14.5)	8.2 (3.4–9.6)	5.8	11.1 (5.9–20.6)	7.8	.082

*Note*: A proportion of the baseline data in this cohort has been previously published (Kho et al.,^14^ PLOS Med 2021). *Missing data*: body temperature for 1 *P. falciparum*; IgM data for 2 *P. falciparum*; white blood cell differential for 1 control, 1 *P. vivax*, and 2 *P. falciparum*; splenic red‐pulp and white‐pulp data for 1 *P. vivax* and two mixed infections. *p*‐values <.05 are bolded.

Abbreviations: IQR, interquartile range; SD, standard deviation; CV, covariance.

^a^ Plasmodium positivity defined as being positive by microscopy and/or polymerase chain reaction.

^b^ Eight patients treated with dihydroartemisinin–piperaquine + single dose of primaquine (two patients 3 days before splenectomy, six patients without timing data) and three patients treated with intravenous artesunate (2, 3, and 9.5 h before splenectomy).

* The Mann–Whitney test was used to compare continuous variables, and the chi‐squared test was used for categorical variables. *p*‐value <.05 was considered statistically significant.

Indications for splenectomy were trauma in 35/37 (95%) and elective surgery due to massively enlarged spleens in 2/37 (5%). Age, gender, and ethnicity between groups were similar except for PCR‐negative individuals, who were younger, all males, and had underrepresentation of Papuans. On admission, 70% (26/37) of patients were anemic (hemoglobin <12 g/dL). Pf‐infected individuals were significantly more anemic and thrombocytopenic than those infected with Pv (Table 1). Plasma IgM concentrations were significantly lower in Pv than in Pf infections (Table 1; Figure S2). None of the 95% of patients (35/37) tested for HIV were seropositive, and none had histopathological evidence of alternative pathology.

### 3.2 Larger spleens in infection with Pf than with Pv

Overall, the median spleen weight was 357 [range: 80–1918] grams. Pf‐infected spleens (median 464 g [range: 144–1918]) were significantly larger than Pv‐infected spleens (median 279 g [range: 80–1250], *p* = .03) and PCR‐negative spleens (median 228 g [range: 112–296], *p* = .02; Figure 1A), including after controlling for body weight (Figure S2). Two individuals with Pf infection had plasma IgM levels two standard deviations (SD) greater than the mean of the Timika population (327 [SD: 385] mg/dL^14^), a diagnostic criterion for hyperreactive malarial splenomegaly (HMS^21^). One Pv‐infected spleen weighing 1.25 kg did not meet criteria for HMS (plasma IgM: 226 mg/dL).

Plasma IgM levels were correlated significantly with spleen weight (Figure S2). This relationship supports malarial splenomegaly as a continuous process leading to HMS, justifying the inclusion of the two HMS cases in our analyses. Spleen weight was also negatively correlated with circulating white blood cell and platelet counts (Figure S2).

### 3.3 RBC‐based congestion and red‐pulp expansion correlate with extent of splenomegaly

Giemsa‐stained spleen sections showed loss of architectural organization with increasing spleen size (Figure 1Bi). A greater proportion of red‐pulp than white‐pulp was universally observed across spleens. The proportion of red‐pulp was 89.5% in Pf‐infected spleens compared to 84.5% in Pv‐infected spleens (*p* = .047; Table 1). There was a strong positive correlation between the proportion of red‐pulp and spleen weight (*r* = .59, *p* = .0003; Figure 1C) and a negative correlation between the proportion of white‐pulp and spleen weight (*r* = −.50, *p* = .002; Figure 1D). Within the red‐pulp, RBC congestion (Figure 1Bii) and dissolution of white‐pulp (Figure 1Bi) were histologically apparent in larger spleens. Quantification of red‐pulp RBCs showed that the number of RBCs/HPF increased with spleen weight in both Pf and Pv infections (*r* = .73, *p* = .017 and *r* = .94, *p* = .006, respectively, Figure 1E). These correlations remained significant after controlling for body weight (Figure S2). Thus, splenomegaly in Pf and Pv infections is associated with expansion of red‐pulp from the congestion of mostly uninfected RBCs, mirroring the relative loss of white‐pulp. Histological evidence of erythrophagocytosis was observed in the red‐pulp (Figure 1Bii). The density of macrophages (CD68^+^ cells) in the red‐pulp was not correlated with spleen weight (*r* = −.06, *p* = .81; Figure 1F), suggesting that the capacity for phagocytic clearance of RBCs does not increase with splenic RBC congestion.

### 3.4 Splenic RBC congestion, spleen size, and anemia

**FIGURE 2:**
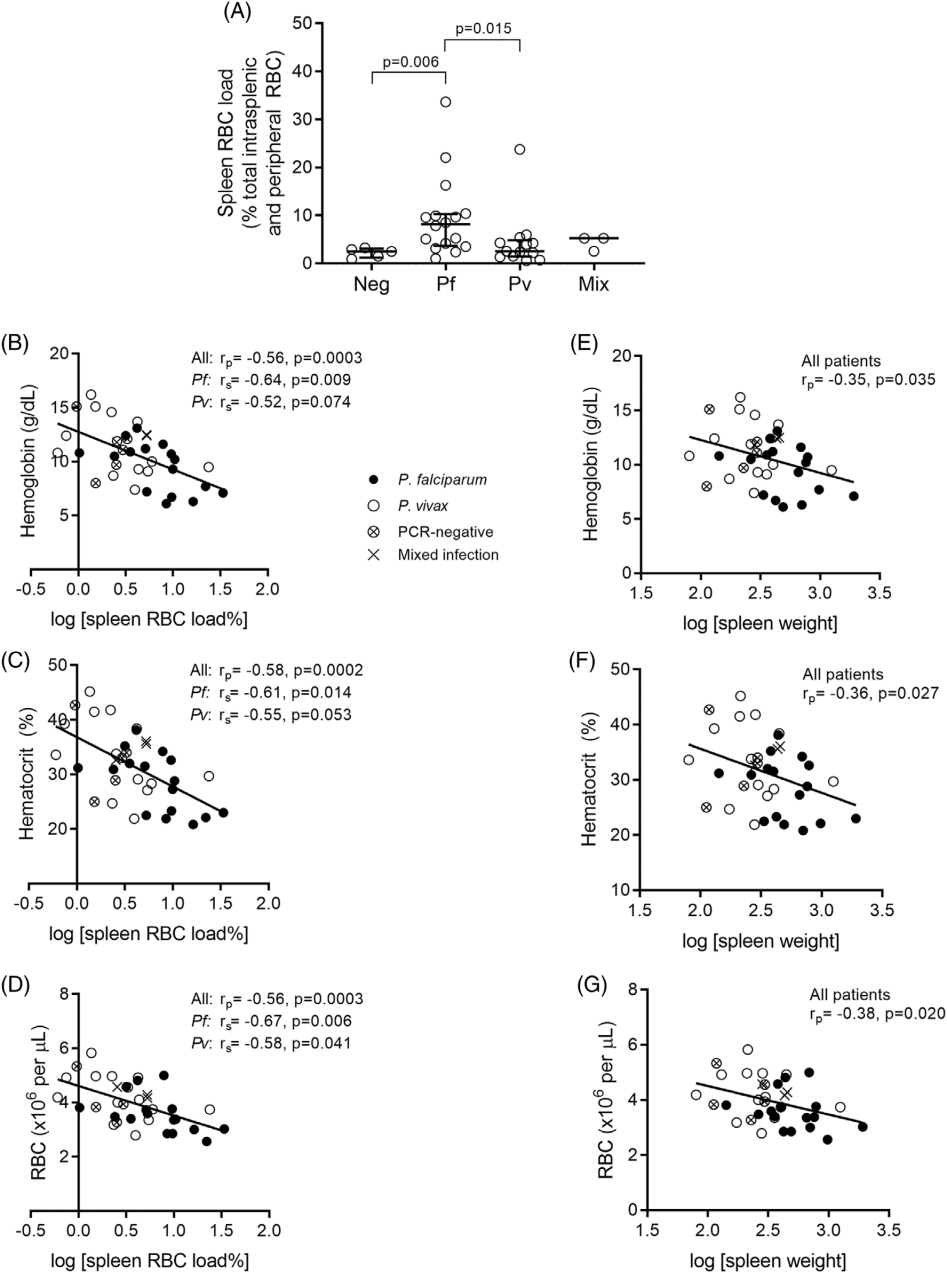
Splenic RBC load and spleen weight correlations with markers of anemia. The proportion of RBCs retained in the spleen was calculated as a percentage of total‐body (intrasplenic and circulating) RBCs for all individuals—16 Pf, 13 Pv, three mixed Pf–Pv, and five PCR‐negative endemic controls (A). These proportions were correlated with markers of anemia including hemoglobin levels (B), hematocrit (C), and circulating RBC counts (D). Spleen weight also correlated with the same circulating markers of anemia (E–G). Correlations were performed as a single group using the Pearson test and for each species using the Spearman test. Spleen weights and RBC loads were log‐transformed and line of best fit was determined by linear regression. RBC, red blood cell; Pf, *Plasmodium falciparum*; Pv, *P. vivax*.

The median estimated proportion of total‐body RBCs retained in Pf‐infected spleens was 8.2% (range: 1.0%–33.6%), significantly higher than the median proportion in Pv (2.6% [range: 0.6%–23.8%], *p* = .015) and PCR‐negative controls (2.5% [range: 1.0%–3.3%], *p* = .006; Figure 2A). The maximum observed proportions were 33.6% in Pf, 23.8% in Pv, and 3.3% in PCR‐negative controls (Figure 2A). The proportion of total‐body RBCs being retained in the spleen was correlated negatively with hemoglobin concentration, hematocrit, and circulating RBC count in both Pf and Pv (Figure 2B–D). Spleen size also correlated negatively with these markers of oxygen‐carrying capacity in circulation (Figure 2E–G).

To estimate the degree to which splenic RBC congestion contributes to malarial anemia in *P. falciparum*, we compared the differences in hemoglobin and splenic RBC load with PCR‐negative endemic controls. The median difference in hemoglobin concentration between Pf infections (10.2 g/dL) and PCR‐negative controls (11.1 g/dL) was 0.9 g/dL, consistent with that reported previously in the wider Timika population.^2^ This corresponds to an 8.1% loss of circulating RBCs in Pf infections. Overall, the difference in splenic RBC load percentage in Pf infections (median load 8.2%) compared to PCR‐negative controls (median load 2.5%) was 5.7%. Using these figures, we estimate that splenic RBC congestion accounted for 70% ([5.7÷8.1] × 100) of circulating RBC loss seen in Pf infections in our cohorts. The median proportion of circulating reticulocytes, measured in the first 11 patients (7 Pf, 4 Pv), was 1.8% (range: 1.1%–7.4%), suggesting that bone marrow RBC production was not reduced. Thus, while splenic RBC congestion comprised a minority of total‐body RBCs, our data suggest this process accounts for the majority of malarial anemia.

A subset of 10/37 (27%) splenectomy patients in our cohorts commenced transfusion a median of 63 (interquartile range: 34–266) mins prior to splenectomy and blood collection (Table 1). Excluding these individuals and analyzing non‐transfused patients alone did not significantly alter our findings (Figures S3 and S4), suggesting that pre‐operative transfusion was not a confounding factor.

### 3.5 RBC deformability, splenic filtration stringency, and spleen size

**FIGURE 3:**
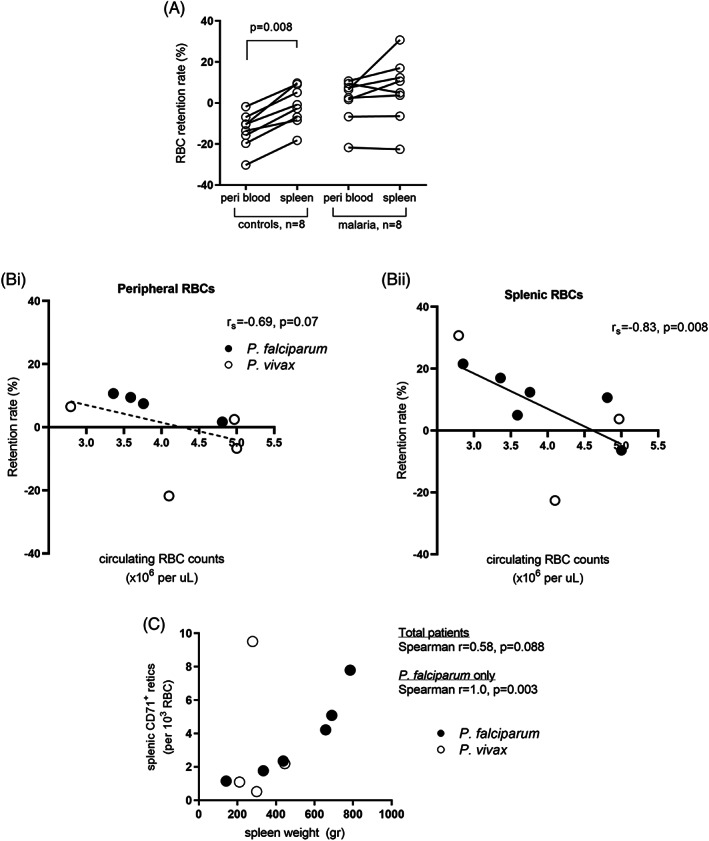
RBC retention rates, anemia, and reticulocytes. The rate of RBC retention was used as a measure of RBC deformability as determined by microsphiltration. The retention rates of RBC populations from paired peripheral blood and spleen blood samples were available for eight individuals with asymptomatic infections and eight non‐endemic controls (A). Paired samples were compared using the Wilcoxon test and unpaired samples using the Mann–Whitney test. Peripheral (Bi) and splenic (Bii) RBC retention rates were correlated with circulating RBC counts. The concentration of stiff immature CD71+ retics was determined by flow cytometry in a subset of 10 individuals (*P. vivax n* = 4, *P. falciparum n* = 6) and was correlated with spleen weight as a marker for rigid cells to investigate splenic biomechanical filtration stringency (C). All correlations were performed using the Spearman test. Lines of best fit in B were generated by linear regression. RBC, red blood cells; retics, reticulocytes.

To determine whether biomechanical trapping of RBCs with reduced deformability contributes to RBC congestion in the spleen, we measured the ex vivo retention rate of splenic and peripheral RBCs by microsphiltration in a subset of nine individuals from Cohort 1 (six Pf and three Pv, eight paired) and in eight non‐endemic controls from France. In non‐endemic controls, retention rates of splenic RBCs were significantly higher compared to peripheral RBCs (*p* = .008, Figure 3A), consistent with greater rigidity of trapped splenic RBCs than those in circulation under normal conditions. In contrast, retention rates of splenic and peripheral RBCs were similar in *Plasmodium* infections (*p* = .11; Figure 3A), consistent with reduced deformability of circulating RBCs in malaria.

In *Plasmodium* infections, RBC retention rates were correlated negatively with circulating RBC counts, with the relationship stronger for splenic RBCs than for peripheral RBCs (Figures 3Bi and 3Bii). Splenic RBC retentions rates were also correlated negatively with hematocrit levels (*r* = −.63, *p* = .078). These data are consistent with a role for reduced RBC deformability in contributing to malarial anemia.

In *Plasmodium* infections, the retention rates of splenic RBCs were not correlated with red‐pulp area (*r* = −.23, *p* = .55), red‐pulp RBC counts per HPF (*r* = −.5, *p* = .27), spleen weight (*r* = .12, *p* = .76), or the proportion of total‐body RBCs retained in the spleen (*r* = .23, *p* = .55), suggesting the presence of additional factors contributing to RBC congestion and splenomegaly, i.e., not increased RBC rigidity alone. We tested the hypothesis that larger spleens display greater stringency at filtering RBCs using the concentration of rigid CD71^+^ reticulocytes^20^ as a plausible marker for splenic biomechanical filtration capacity. As hypothesized, immature CD71^+^ reticulocyte counts in spleen blood correlated positively with spleen weight (*r* = .58, *p* = .088; Figure 3C). When categorizing spleens by *Plasmodium* infection, this relationship was highly significant in Pf (r = 1.0, *p* = .003, *n* = 6) but not apparent in Pv infection (*n* = 4, Figure 3C). These correlations were also apparent when immature reticulocytes were categorized further into CD71^low^, CD71^intermediate^, and CD71^high^ subsets (Figure S5). In contrast, splenic blood counts of deformable CD71^−^ mature reticulocytes were not correlated with spleen weight (Figure S5). Controlling for the degree of anemia (circulating RBC counts) did not alter our findings significantly (Table S2), indicating that any reticulocytosis occurring in response to the anemia in larger spleens was not a confounding factor.

## 4 DISCUSSION

Our study highlights that splenic congestion with mostly uninfected RBC accounts for malarial splenomegaly and is likely the most important mechanism of malarial anemia. The opportunity to analyze removed spleens, both macroscopically and microscopically, generated an unprecedently precise set of observations. Spleen weight is a more relevant measure of spleen size than clinical palpation or imaging and was correlated with congestion‐defining measures, including with the proportion of red‐pulp and with the density of RBC on histology. In Pf‐infected subjects, RBCs retained in spleens accounted for more than half of the circulating RBC loss. Moreover, splenic CD71‐positive reticulocyte concentrations correlated with spleen weight and production of circulating reticulocytes was maintained. Therefore, impaired RBC production was a minor component of anemia in this cohort. In a subset of patients, microsphiltration of RBC directly confirmed that splenic biomechanical sensing of RBC is altered during and after malaria^22^, ^23^ and that retention rates of both peripheral and splenic RBCs were correlated with anemia. Taken together, these quantitative observations indicate that excessive spleen‐related RBC loss is the predominant cause of malarial anemia, as previously suggested by ex vivo perfusion of human spleens.^24^

Splenic congestion with uninfected RBCs has been reported qualitatively in acute fatal falciparum malaria.^25^, ^26^, ^27^, ^28^, ^29^ We now show quantitatively that this mechanism is predominant in both chronic asymptomatic and acute uncomplicated infections. RBC congestion is associated with expansion of the red‐pulp and is the key mechanism underlying splenic enlargement in both Pf and Pv. We estimate that up to 33.6% of total RBCs in Pf and 23.8% in Pv are retained in the spleen and are associated with reductions in hemoglobin concentrations and hematocrit, reflecting an apparent loss of RBCs in circulation. These estimates are consistent with a radioisotope study in malaria‐endemic New Guinea reporting splenic pooling of 3%–38% of RBCs.^30^ In malaria‐endemic Uganda and Malawi, splenic enlargement increased the risk of anemia in children who were re‐admitted compared to those without splenomegaly.^9^ In Ugandan and Sudanese studies, splenomegaly was also more prevalent in children with severe malarial anemia,^10^, ^11^, ^12^ with spleen size inversely correlated with hemoglobin.^12^ Splenomegaly also occurs in chronic malaria and can lead to HMS, a syndrome characterized by massively enlarged spleens, hypersplenism, and high polyclonal IgM titers following long‐term parasite exposure.^31^ Anemia is almost universally present in HMS cases,^32^, ^33^ with one HMS case in our cohort having the largest spleen and greatest degree of splenic congestion and anemia.

Splenic filtration of RBC occurs in the macrophage‐rich cords of the red‐pulp and at tight interendothelial slits allowing passage only to deformable cells from the red‐pulp cords to venous sinuses.^34^ While there is constant exchange of RBCs from the splenic pool and circulation,^35^ sustained trapping of a proportion of uninfected RBCs in the spleen, as well as loss of infected‐RBCs from a hidden asexual endosplenic lifecycle,^13^ likely further contributes to the anemia seen in asymptomatic infections.^2^ We had previously shown that parasites can be cultured from splenic blood.^13^ There is enhanced RBC clearance following treatment in uncomplicated^36^ and severe falciparum malaria^37^ and following parasite clearance.^38^, ^39^ Enhanced RBC clearance also occurs in symptomatic malaria patients with splenomegaly but not in those without.^23^ Post‐artesunate delayed hemolysis (PADH) was an unlikely contributor to anemia in this study. In the 11 patients (30% of the cohort) who had received prior antimalarial therapy, the timing of its administration and the high level of preexisting antimalarial immunity in the area^40^, ^41^ each argue against the occurrence of PADH‐related anemia at the time of splenectomy. We show that blood from spleens contains higher proportions of rigid RBC in anemic subjects, suggesting that splenic retention of rigid RBC contributes to anemia. Our correlative analyses of weight‐dependent retention of stiff CD71+ immature reticulocytes in falciparum malaria also provide evidence of increased splenic filtration stringency in larger spleens. While studies in greater numbers of subjects are needed to clarify the magnitude of additional filtration stringency and its role in splenic RBC retention in malaria, the findings are consistent with the accelerated splenic clearance of stiff ^51^Cr‐labeled RBCs previously observed in Thai Pf‐infected patients with splenomegaly.^23^ Such conclusions are not limited to studies in malaria. Enhanced splenic RBC sequestration is seen in splenomegaly due to cirrhosis and other causes, supporting the concept that increased spleen size in itself can result in increased filtration stringency^42^ regardless of the underlying cause of congestive splenomegaly.

Beyond biomechanical retention, several immune‐related processes may contribute to uninfected RBC congestion,^43^, ^44^, ^45^, ^46^, ^47^, ^48^ with or without accelerated macrophage clearance. The intensity of splenic congestion observed in our cohort indicates that splenic retention of uninfected RBC exceeds their elimination, as recently suspected in sickle cell disease.^49^ Further work is needed to better understand the mechanisms tuning this equilibrium in malaria.

Larger spleens in Pf than in Pv infections suggest a greater retention of uninfected RBCs in the red‐pulp cords, possibly from the greater reduction in deformability of uninfected RBCs apparent in falciparum malaria^50^, ^51^ or from greater splenic filtration stringency in Pf infection.^22^ With Pf capable of infecting RBCs of all ages, the greater retention and congestion of RBCs provide an abundance of target cells favoring the recently identified endosplenic Pf lifecycle.^13^ Our findings also challenge the assumptions underlying prevailing models of malarial anemia and the proportion of uninfected‐to‐infected erythrocytes lost in infection with both *Plasmodium* spp.,^3^, ^52^, ^53^, ^54^ where all parasites have been previously assumed to circulate. The hidden endosplenic lifecycles of parasitized RBC,^13^ particularly with Pv,^14^ suggest that the latter is not the case.

Our study has several limitations. We estimated the splenic RBC loss from circulation based on splenic RBC retention at a single time‐point (splenectomy), which does not account for removal by macrophage erythrophagocytosis. Data from microfluidic and spleen perfusion models indicate that RBC engulfment by splenic macrophages occurs rapidly,^49^ suggesting rapid turnover of congested RBCs and thus a significant underestimation of splenic RBC retention and loss in our cohort. This suggests that our estimates of splenic RBC retention are conservative and may in fact make an even greater contribution to anemia. Pre‐splenectomy transfusion in a quarter of our patients may have confounded the relationships between splenic RBC congestion and circulating measures of RBC loss. However, the same relationships were also found in the majority without transfusion. Furthermore, our finding that an increase in the proportion of total‐body RBCs being retained in spleen was negatively correlated with hemoglobin levels, hematocrit, and circulating RBC counts suggests that observations were unrelated to trauma‐related anemia. While the splenectomized population may not be wholly representative of the population at large, multiple studies in the general malaria‐exposed population have shown a robust correlation between splenomegaly and malarial anemia.^9^, ^10^, ^11^, ^12^, ^30^, ^31^, ^32^, ^33^ We were unable to investigate the role of genetics on splenic RBC retention, clearance, and splenomegaly in our mixed cohort of Papuans and non‐Papuans, processes recently shown to be genetically controlled in Fulani people.^22^

In conclusion, our evaluation of human spleens from a prospective splenectomy series in a malaria‐endemic region provides novel quantifications supporting splenic RBC congestion and expansion of red‐pulp as key underlying mechanisms of malarial anemia and splenomegaly. Congestion of RBCs in the spleen has detrimental consequences including anemia and potential white‐pulp dysfunction. Our data indicate that the spleen threshold changes during malaria, whereby retention of a rigid RBC likely results in retention of 2–3 additional RBCs. How congestion, stringency, and anemia are interrelated, including hierarchy in their causal–effect relationship, deserves further study.

## AUTHOR CONTRIBUTIONS

PAB and NMA conceived the study; SK, NCS, LQ, AF, AS, DH, and PAB provided methodology; SK, AF, AS, DH, and PAB performed validation; SK, LQ, AF, AS, PAIS, FC, NNK, HR, BA, DH, NIM, FF, DAR, AMP, LL, BTGY, DSM, and PP performed experiments and investigation; SK, NCS, PAIS, LT, EK, RNP, TWY, GM, RN, JRP, NMA, and PAB provided resources; SK, NMA, and PAB performed formal analysis and wrote the original draft; AS, GM, RNP, and TWY performed review and editing; SK performed visualization; SK, NCS, LT, EK, RN, JRP, NMA, and PAB provided supervision; SK, NCS, RN, JRP, NMA, and PAB performed project administration; and SK, RNP, JRP, NMA, and PAB provided funding acquisition.

## FUNDING INFORMATION

The study was supported by the Australian National Health and Medical Research Council (Program Grant No. 1037304 and Fellowship Nos. 1042072 and 1135820 to NMA, Ideas Grant 2019153 to SK, Investigator Grant 2008501 to RNP [Grant No. 1131932] from Improving Health Outcomes in the Tropical North, and the Australian Centre of Research Excellence in Malaria Elimination), by a grant from the Institut National de la Santé et de la Recherche Médicale to PAB, by a grant from the Wellcome Trust (no. 099875) to JRP, by an Australian Government Postgraduate Award Scholarship to SK, and by the Australian Department of Foreign Affairs and Trade.

## CONFLICT OF INTEREST STATEMENT

The authors declare that no competing interest exists.

## Supporting information


**Figure S1.** Flow cytometry gating of patient RBC populations in the microsphiltration assay. Upstream and downstream samples containing PKH‐labeled and unlabeled RBC populations from patients and controls were acquired on a BD Accuri C6 flow cytometer and gated as shown. After gating on singlets and RBCs, PKH‐26‐labeled patient RBC populations (peripheral or spleen) were gated based on positivity in the FL2 channel and their retention rates were calculated (see the “Methods” section). Abbreviations: RBC, red blood cell.

**Figure S2.** Plasma IgM, pancytopenia, and normalized spleen weight. Total plasma IgM concentrations were measured on a Siemens BN‐ProSpec analyzer in five controls, 14 Pf, 13 Pv, and three mixed Pf–Pv infections (A). Spleen weight normalized to bodyweight was compared in five PCR‐negative endemic controls, 16 Pf, 13 Pv, and three mixed Pf–Pv infections (B). Individual datapoints, medians, and interquartile ranges are shown and compared using the Mann–Whitney test. Total plasma IgM concentrations (C), automated WBC counts (D), and platelet counts (E) were correlated with spleen weight using the Pearson test following log transformation. Correlations in Figure 1 were repeated using normalized spleen weight (corrected to body weight) including the area of red‐pulp, white‐pulp, and the number of RBCs per red‐pulp field of view (F). Abbreviation: Pf, *Plasmodium falciparum*; Pv, *Plasmodium vivax*; IgM, immunoglobulin M; WBC, white blood cells.

**Figure S3.** Spleen weight, red‐pulp expansion, and RBC congestion in non‐transfused splenectomy patients. Spleen weight (in grams) was grouped in non‐transfused patients according to infecting Plasmodium species comprising of 12 with Pf, nine with Pv, three mixed Pf–Pv infections, and three PCR‐negative endemic controls (A). Irrespective of infecting species, spleen weights were similar between patients receiving transfusion prior to sampling (*n* = 10) and non‐transfused patients (*n* = 27) (B). Correlations between spleen weight and the proportion of red‐pulp (C), and spleen weight and the proportion of white‐pulp (D) were determined histologically. The number of RBCs per red‐pulp field of view in the non‐transfused subset of Pf‐infected (*n* = 7) and Pv‐infected spleens (*n* = 4) were correlated with spleen weight (D). The number of macrophages (CD68+ cells) per unit volume of red‐pulp was also counted in the non‐transfused subset of spleens (*n* = 14) and correlated with spleen weight (F). Median, interquartile range, and individual datapoints are shown in A and B. The Mann–Whitney test was used for statistical comparison. In C–E, spleen weights were log‐transformed and line of best fit was determined by linear regression. Correlations were examined using the Pearson (r_s_) or Spearman (r_p_) test. A *p*‐value <.05 was considered significant. Abbreviations: Pf, *Plasmodium falciparum*; Pv, *Plasmodium vivax*; RBC, red blood cell; PCR, polymerase chain reaction.

**Figure S4.** Splenic RBC load and correlations with markers of anemia in non‐transfused splenectomy patients. The proportion of RBCs retained in the spleen was evaluated in non‐transfused splenectomy patients—12 Pf, 9 Pv, three mixed Pf–Pv, and three PCR‐negative endemic controls (A). These proportions were correlated with markers of anemia including hemoglobin levels (B), hematocrit (C), and circulating RBC counts (D). Correlations were performed as a single group using the Pearson test. Spleen RBC loads were log‐transformed, and line of best fit was determined by linear regression. Abbreviations: RBC, red blood cell; Pf, *Plasmodium falciparum*; Pv, *P. vivax*.

**Figure S5.** Splenic reticulocytes. Immature CD71+ retic concentrations in the spleen were determined by flow cytometry in a subset of 10 individuals (*P. vivax n* = 4, *P. falciparum n* = 6) and used as a marker for rigid cells to investigate splenic biomechanical filtration stringency. Immature CD71+ retics were categorized into subgroups of CD71‐low, CD71‐intermediate, and CD71‐high expression as previously described (Kho et al, PLOS Med, 2021) and were each correlated with spleen weight (A). The concentration of CD71‐mature retics (B) was also correlated with spleen weight (B). Correlations were performed using the Spearman test. RBC, red blood cell; retic, reticulocyte.

**Table S1.** Spleen RBC volume and spleen weight in four normal spleens.
**Table S2.** Correlations between splenic CD71^+^ reticulocytes and spleen weight.

## Data Availability

Data in this manuscript are available from the corresponding author upon reasonable request.
