# Supplementary figures and images for: Retention of uninfected red blood cells causing congestive splenomegaly is the major mechanism of anemia in malaria
Source: Am J Hematol. 2023 Nov 27;99(2):223–35. doi: 10.1002/ajh.27152 (PMC10952982; doi:10.1002/ajh.27152)

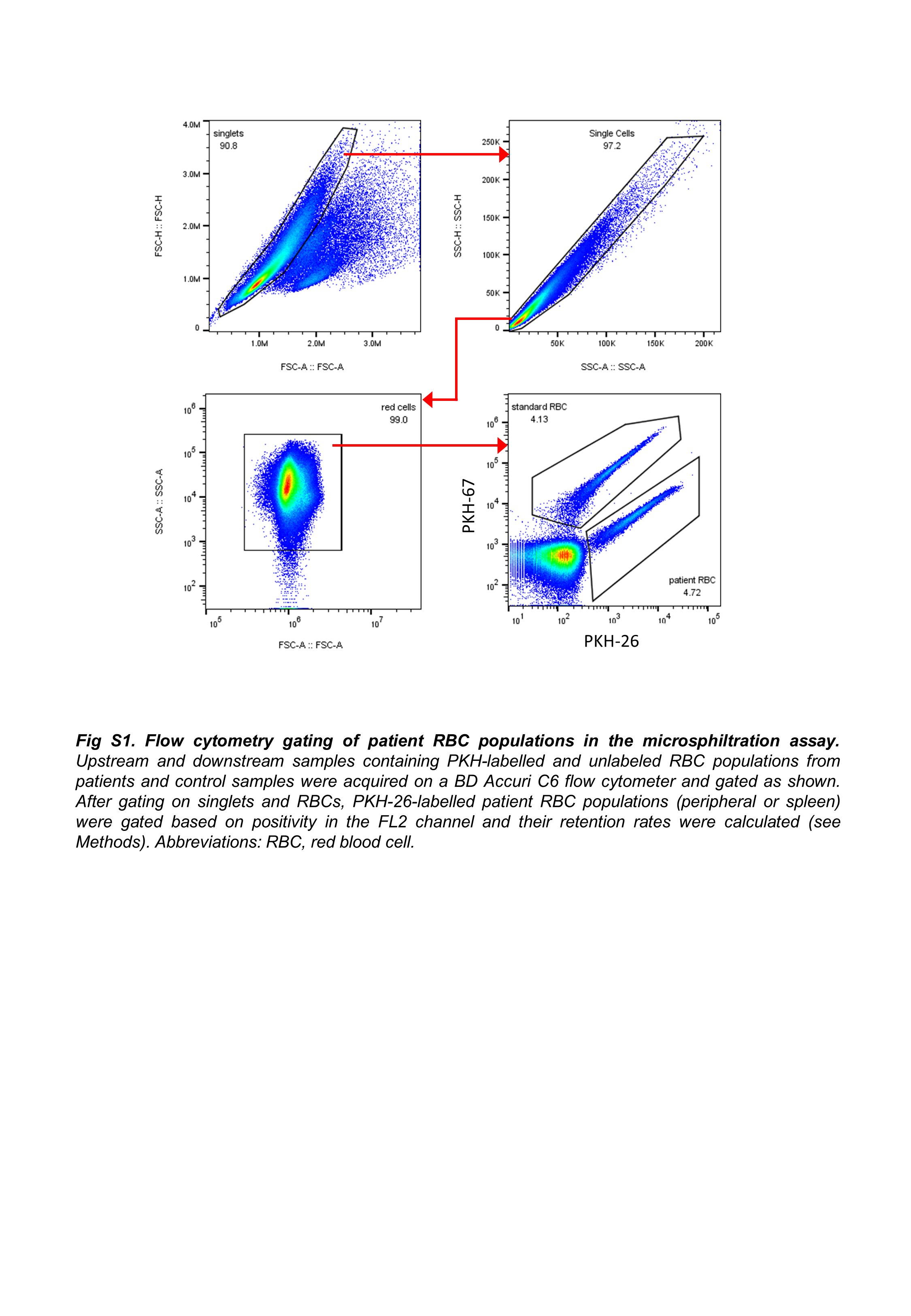

Supplement: Supplementary file 1 — Figure S1. Flow cytometry gating of patient RBC populations in the microsphiltration assay. Upstream and downstream samples containing PKH‐labeled and unlabeled RBC populations from patients and controls were acquired on a BD Accuri C6 flow cytometer and gated as shown. After gating on singlets and RBCs, PKH‐26‐labeled patient RBC populations (peripheral or spleen) were gated based on positivity in the FL2 channel and their retention rates were calculated (see the “Methods” section). Abbreviations: RBC, red blood cell. [file AJH-99-223-s006.TIF]

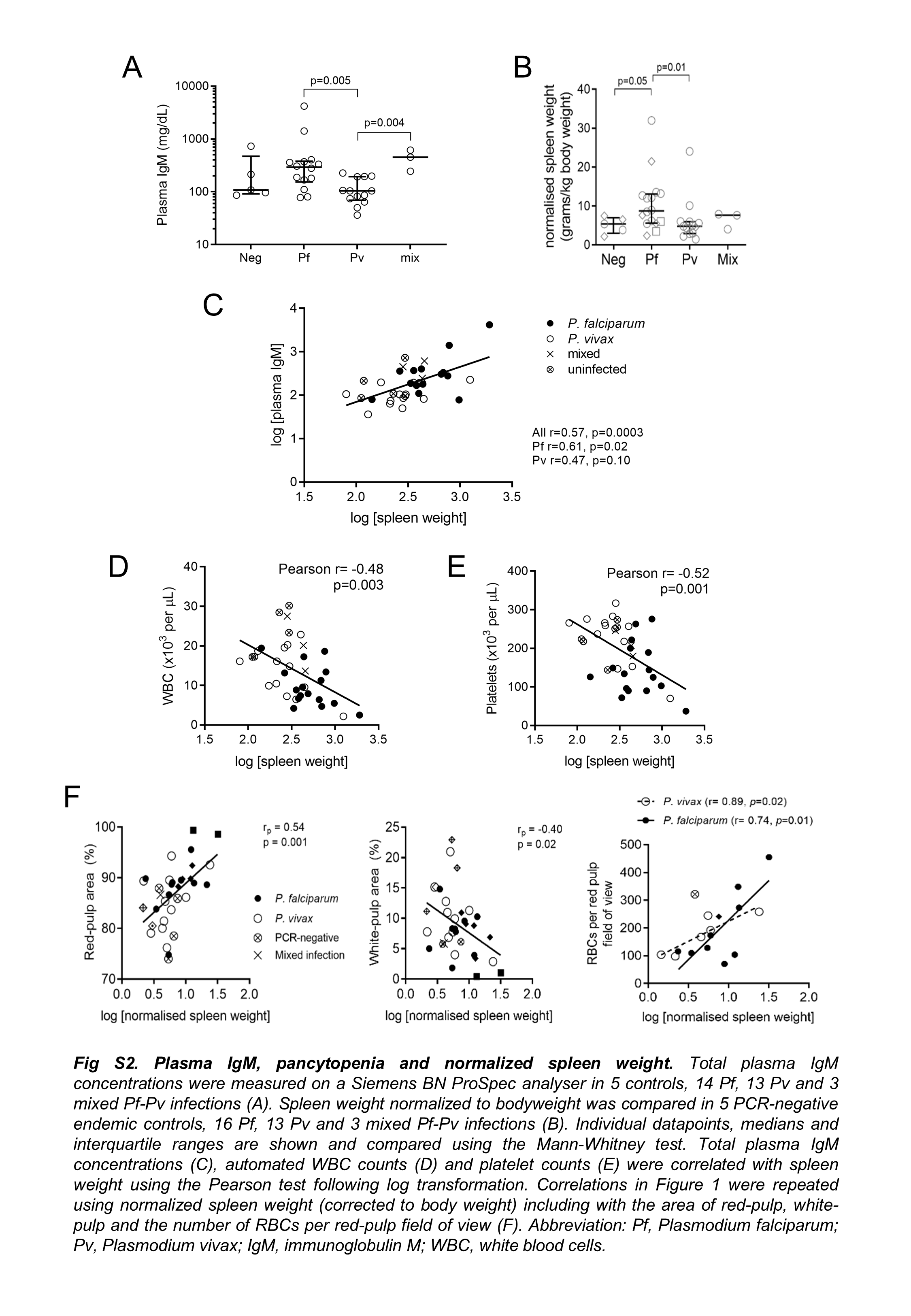

Supplement: Supplementary file 2 — Figure S2. Plasma IgM, pancytopenia, and normalized spleen weight. Total plasma IgM concentrations were measured on a Siemens BN‐ProSpec analyzer in five controls, 14 Pf, 13 Pv, and three mixed Pf–Pv infections (A). Spleen weight normalized to bodyweight was compared in five PCR‐negative endemic controls, 16 Pf, 13 Pv, and three mixed Pf–Pv infections (B). Individual datapoints, medians, and interquartile ranges are shown and compared using the Mann–Whitney test. Total plasma IgM concentrations (C), automated WBC counts (D), and platelet counts (E) were correlated with spleen weight using the Pearson test following log transformation. Correlations in Figure 1 were repeated using normalized spleen weight (corrected to body weight) including the area of red‐pulp, white‐pulp, and the number of RBCs per red‐pulp field of view (F). Abbreviation: Pf, Plasmodium falciparum; Pv, Plasmodium vivax; IgM, immunoglobulin M; WBC, white blood cells. [file AJH-99-223-s003.TIF]

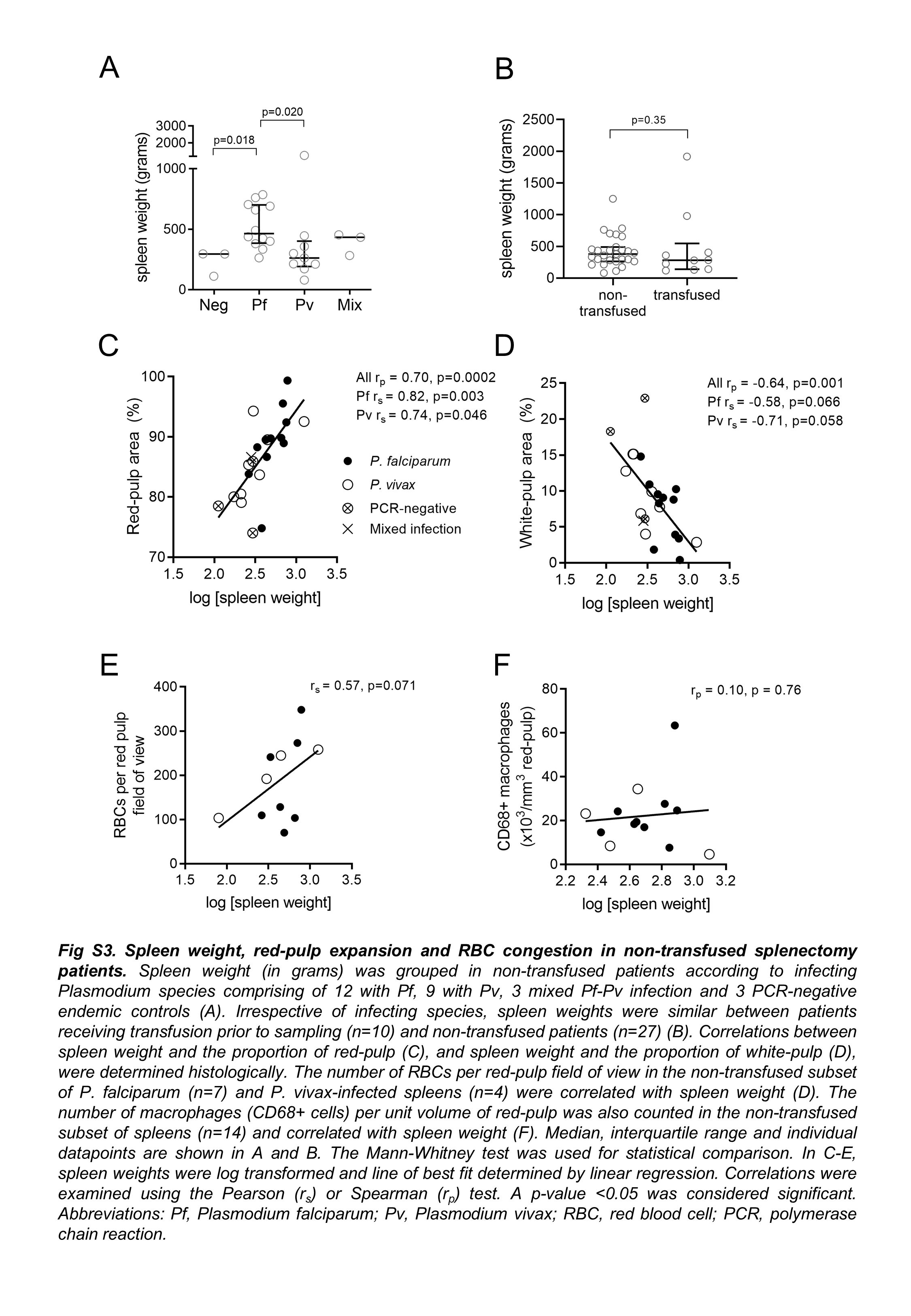

Supplement: Supplementary file 3 — Figure S3. Spleen weight, red‐pulp expansion, and RBC congestion in non‐transfused splenectomy patients. Spleen weight (in grams) was grouped in non‐transfused patients according to infecting Plasmodium species comprising of 12 with Pf, nine with Pv, three mixed Pf–Pv infections, and three PCR‐negative endemic controls (A). Irrespective of infecting species, spleen weights were similar between patients receiving transfusion prior to sampling (n = 10) and non‐transfused patients (n = 27) (B). Correlations between spleen weight and the proportion of red‐pulp (C), and spleen weight and the proportion of white‐pulp (D) were determined histologically. The number of RBCs per red‐pulp field of view in the non‐transfused subset of Pf‐infected (n = 7) and Pv‐infected spleens (n = 4) were correlated with spleen weight (D). The number of macrophages (CD68+ cells) per unit volume of red‐pulp was also counted in the non‐transfused subset of spleens (n = 14) and correlated with spleen weight (F). Median, interquartile range, and individual datapoints are shown in A and B. The Mann–Whitney test was used for statistical comparison. In C–E, spleen weights were log‐transformed and line of best fit was determined by linear regression. Correlations were examined using the Pearson (rs) or Spearman (rp) test. A p‐value <.05 was considered significant. Abbreviations: Pf, Plasmodium falciparum; Pv, Plasmodium vivax; RBC, red blood cell; PCR, polymerase chain reaction. [file AJH-99-223-s005.TIF]

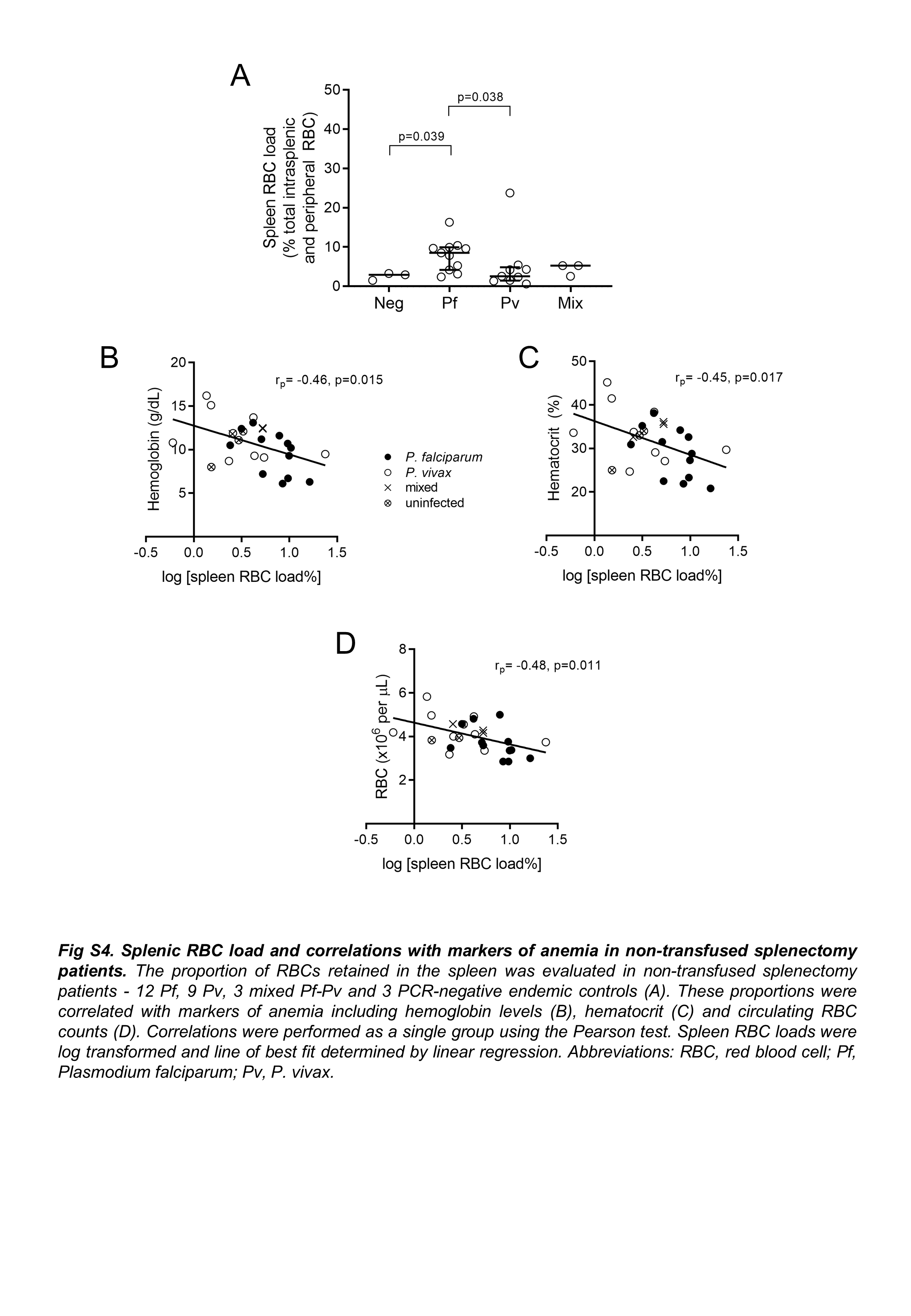

Supplement: Supplementary file 4 — Figure S4. Splenic RBC load and correlations with markers of anemia in non‐transfused splenectomy patients. The proportion of RBCs retained in the spleen was evaluated in non‐transfused splenectomy patients—12 Pf, 9 Pv, three mixed Pf–Pv, and three PCR‐negative endemic controls (A). These proportions were correlated with markers of anemia including hemoglobin levels (B), hematocrit (C), and circulating RBC counts (D). Correlations were performed as a single group using the Pearson test. Spleen RBC loads were log‐transformed, and line of best fit was determined by linear regression. Abbreviations: RBC, red blood cell; Pf, Plasmodium falciparum; Pv, P. vivax. [file AJH-99-223-s001.TIF]

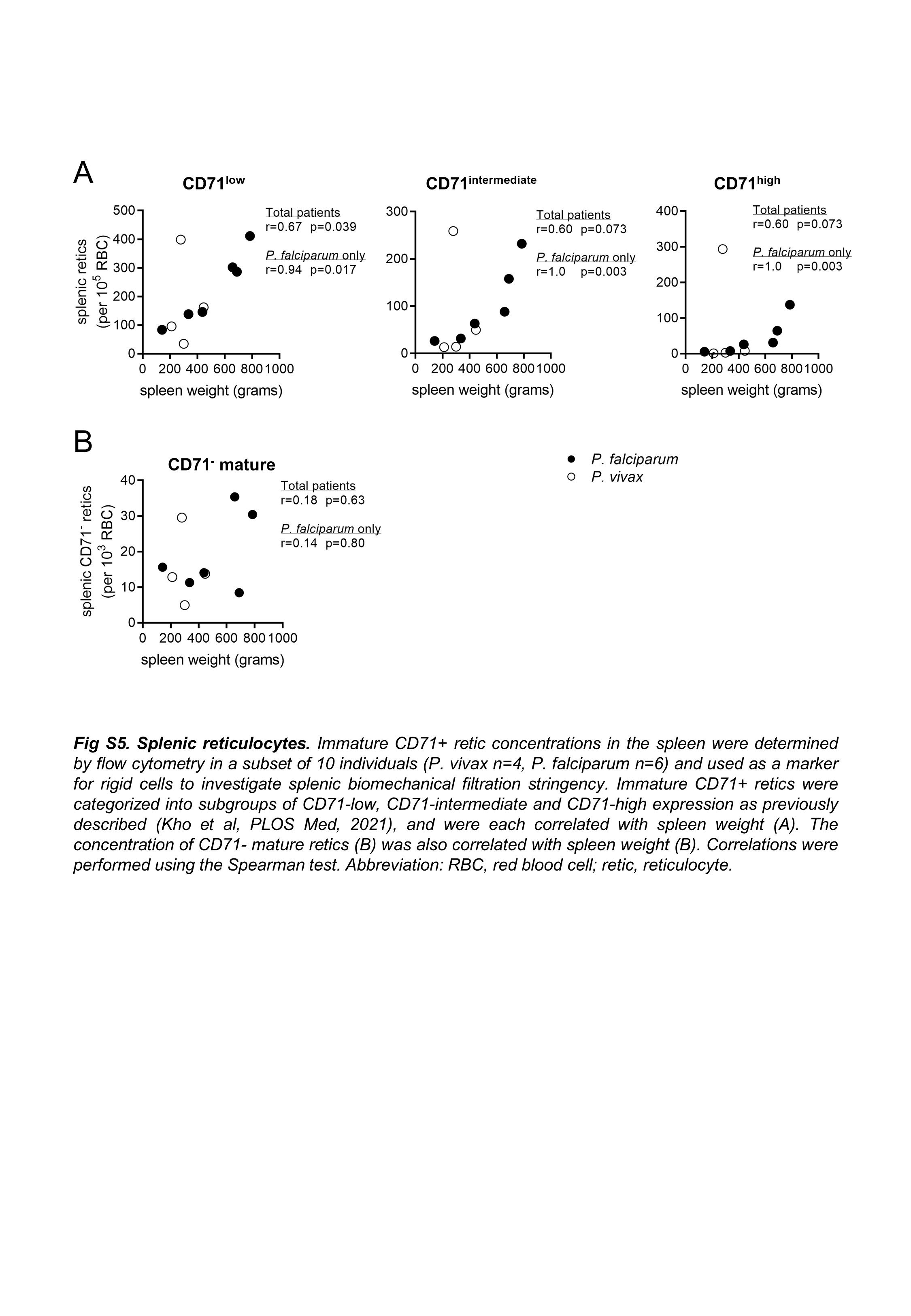

Supplement: Supplementary file 5 — Figure S5. Splenic reticulocytes. Immature CD71+ retic concentrations in the spleen were determined by flow cytometry in a subset of 10 individuals (P. vivax n = 4, P. falciparum n = 6) and used as a marker for rigid cells to investigate splenic biomechanical filtration stringency. Immature CD71+ retics were categorized into subgroups of CD71‐low, CD71‐intermediate, and CD71‐high expression as previously described (Kho et al, PLOS Med, 2021) and were each correlated with spleen weight (A). The concentration of CD71‐mature retics (B) was also correlated with spleen weight (B). Correlations were performed using the Spearman test. RBC, red blood cell; retic, reticulocyte. [file AJH-99-223-s004.TIF]
