# Supplementary material for: Retention of uninfected red blood cells causing congestive splenomegaly is the major mechanism of anemia in malaria
Source: Am J Hematol. 2023 Nov 27;99(2):223–35. doi: 10.1002/ajh.27152 (PMC10952982; doi:10.1002/ajh.27152)
Supplement: Supplementary file 6 — Table S1. Spleen RBC volume and spleen weight in four normal spleens. Table S2. Correlations between splenic CD71+ reticulocytes and spleen weight. [file AJH-99-223-s002.docx]

## Table S1. Spleen RBC volume and spleen weight in 4 normal spleens

| **Patient ID** | **spleen weight (g)** | **spleen RBC**  **volume (mL)** | **spleen RBC volume**  **per 100g tissue (mL)** |
| --- | --- | --- | --- |
| RH93 | 170 | 14 | 8.2 |
| RH95 | 70 | 9.5 | 13.6 |
| RH96 | 130 | 7.5 | 5.8 |
| RH98 | 170 | 9.4 | 5.5 |
| mean | 135.0 | 10.1 | 8.3 |
| SD | 47.3 | 2.8 | 3.7 |

Footnotes:

Abbreviations: RBC, red blood cell; SD, standard deviation.

## Table S2. Correlations between splenic CD71^+^ reticulocytes and spleen weight

| Spleen weight vs | Patient group | Spearman | | pcorr (controlling for RBC counts) | |
| --- | --- | --- | --- | --- | --- |
|  |  | r | p-value | r | p-value |
| splenic CD71^+^ reticulocytes | All patients, n=10 | 0.58 | 0.088 | 0.61 | 0.082 |
| splenic CD71^+^ reticulocytes | *P. falciparum*, n=6 | 1.0 | 0.003 | 0.93 | 0.023 |
| splenic CD71^low^ reticulocytes | All patients, n=10 | 0.67 | 0.039 | 0.78 | 0.014 |
| splenic CD71^low^ reticulocytes | *P. falciparum*, n=6 | 0.94 | 0.017 | 0.98 | 0.004 |
| splenic CD71^int^ reticulocytes | All patients, n=10 | 0.60 | 0.073 | 0.61 | 0.083 |
| splenic CD71^int^ reticulocytes | *P. falciparum*, n=6 | 1.0 | 0.003 | 0.88 | 0.051 |
| splenic CD71^hi^ reticulocytes | All patients, n=10 | 0.60 | 0.073 | 0.23 | 0.55 |
| splenic CD71^hi^ reticulocytes | *P. falciparum*, n=6 | 1.0 | 0.003 | 0.80 | 0.10 |
